# Supplementary material for: The causal role of circulating inflammatory markers in osteoporosis: a bidirectional Mendelian randomized study
Source: Front Immunol. 2024 Jul 18;15:1412298. doi: 10.3389/fimmu.2024.1412298 (PMC11291241; doi:10.3389/fimmu.2024.1412298)
Supplement: Supplementary file 1 [file DataSheet_1.zip › supplementary material/CIM-OSTEOPOROSIS /GCST90274810.csv_leave_one_out.pdf]

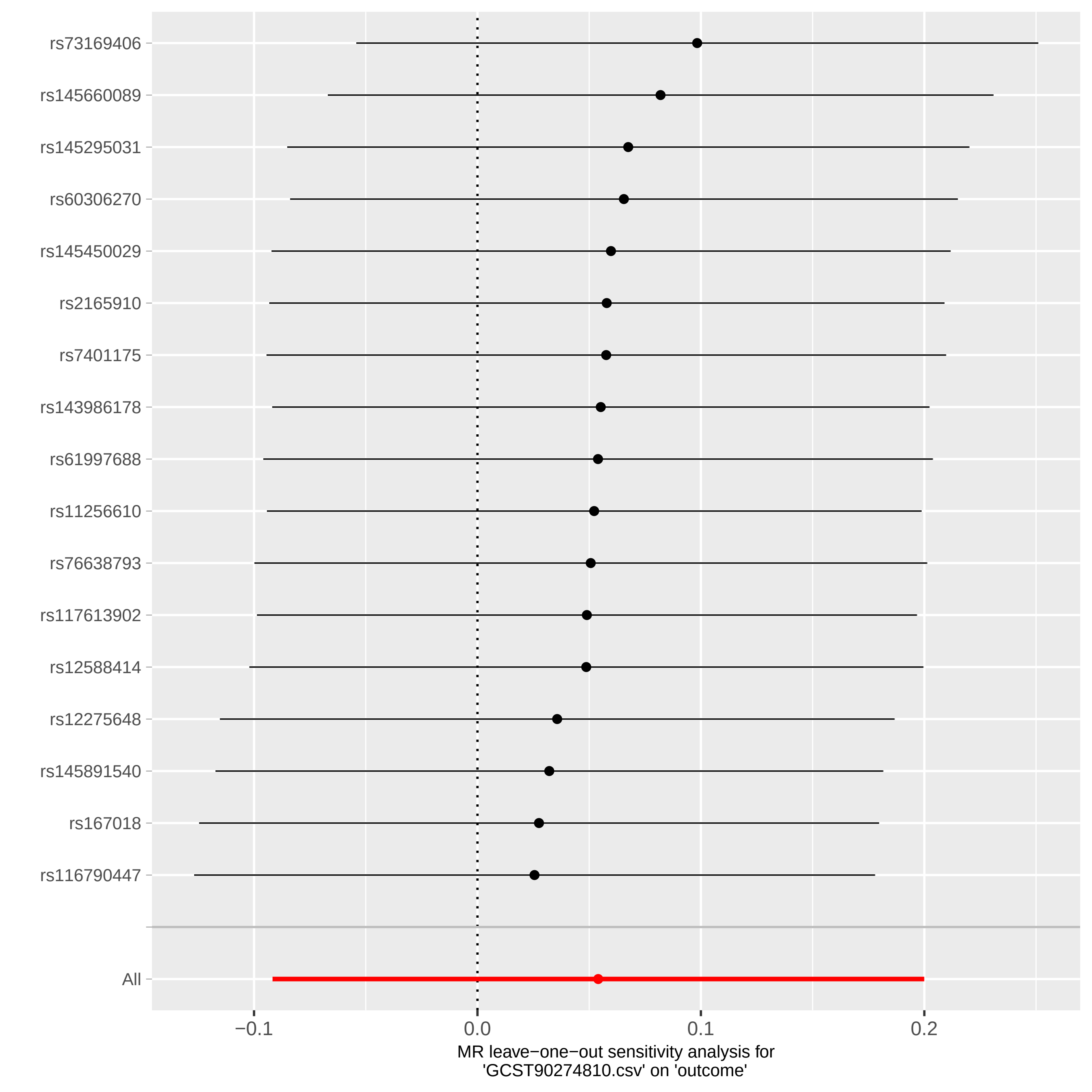
rs73169406
rs145660089
rs145295031
rs60306270
rs145450029
rs2165910
rs7401175
rs143986178
rs61997688
rs11256610
rs76638793
rs117613902
rs12588414
rs12275648
rs145891540
rs167018
rs116790447
All
−0.1
0.0
0.1
0.2
MR leave−one−out sensitivity analysis for
'GCST90274810.csv' on 'outcome'
